# Synthesis of the Sulphonate and Phosphonate Derivatives of Mercaptoacetyltriglycine. X-Ray Crystal Structure of Na_2_[ReO(Mercaptoacetylglycylglycylaminomethanesulphonate)]·3H_2_O

**DOI:** 10.1155/MBD.1994.31

**Published:** 1994

**Authors:** Lory Hansen, Andrew Taylor, Luigi G. Marzilli

**Affiliations:** 1 Departments of Chemistry and Radiology Emory University Atlanta Georgia 30322 USA

## Abstract

Mercaptoacetyltriglycine forms complexes with ^186/188^Re and ^99m^Tc radionuclides that are useful in nuclear medicine because they are substrates of the renal anion transport system.  However, the renal clearance of [MO(MAG_3_)]^2-^(MAG_3_ = penta-anionic form of mercaptoacetyltriglycine, M = Re, Tc) complexes are less than ideal. Organic sulphonates are also transported by the renal anion transport system and phosphonates are similar to sulphonates in size and shape. In an effort to develop new ligands that form Re and Tc complexes and have improved renal clearances compared to [MO(MAG_3_)]^2-^ complexes, the sulphonate and phosphonate derivatives of mercaptoacetyltriglycine were synthesized. The dianion [ReO(MAG_2_-AMS)]^2-^ (MAG_2_-AMS = penta-anionic form of mercaptoacetylglycylglycylaminomethanesulphonic acid) was prepared for characterization by exchange reaction of ReOCl_3_(Me_2_S)(OPPh_3_) and isolated as the disodium salt. The structure of Na_2_[ReO(MAG_2_-AMS)]·3H_2_O (6) was determined by X-ray diffraction. The coordination geometry is pseudo square pyramidal, with the nitrogen and sulfur donor atoms forming a square base and the oxo ligand at the apex. The deprotonated sulphonate group has a syn conformation with respect to the oxo ligand. The renal clearances of [^99m^TcO(MAG_2_-AMS)]^2-^ and [^99m^TcO(MAG_2_-AMP)]^3-^ were similar in rats and suggest that the difference in total charge between the SO_3_^-^ and PO_3_^2-^ groups is not important to renal clearance. However, their renal clearances were 40-50% less than that of [^99m^TcO(MAG_3_)]^2-^ suggesting that the size and shape of the large tetrahedral SO_3_^-^ and PO_3_^2-^ groups of [^99m^TcO(MAG_2_-AMS)]^2-^ and [^99m^TcO(MAG_2_-AMP)]^3-^ inhibit recognition by the renal transport system compared to the small planar CO_2_^-^ group of [^99m^TcO(MAG_3_)]^2-^.


### SYNTHESIS OF THE SULPHONATE AND PHOSPHONATE DERIVATIVES OF MERCAPTOACETYLTRIGLYCINE. X-RAY CRYSTAL STRUCTURE OF $Na_2$[ReO(MERCAPTOACETYLGLYCYLGLYCYL-AMINOMETHANESULPHONATE)]·$3H_2O$

Lory Hansen,* Andrew Taylor, Jr., and Luigi G. Marzilli

Departments of Chemistry and Radiology, Emory University, Atlanta, Georgia 30322, USA

**Abstract**

Mercaptoacetyltriglycine forms complexes with $^{186/188}Re$ and $^{99m}Tc$ radionuclides that are useful in nuclear medicine because they are substrates of the renal anion transport system. However, the renal clearance of $[MO(MAG_3)]^{2-}$ ($MAG_3$ = penta-anionic form of mercaptoacetyltriglycine, M = Re, Tc) complexes are less than ideal. Organic sulphonates are also transported by the renal anion transport system and phosphonates are similar to sulphonates in size and shape. In an effort to develop new ligands that form Re and Tc complexes and have improved renal clearances compared to $[MO(MAG_3)]^{2-}$ complexes, the sulphonate and phosphonate derivatives of mercaptoacetyltriglycine were synthesized. The dianion $[ReO(MAG_2\text{-}AMS)]^{2-}$ ($MAG_2$-AMS = penta-anionic form of mercaptoacetylglycylglycylaminomethanesulphonic acid) was prepared for characterization by exchange reaction of $ReOCl_3(Me_2S)(OPPh_3)$ and isolated as the disodium salt. The structure of $Na_2[ReO(MAG_2\text{-}AMS)]\cdot 3H_2O$ (6) was determined by X-ray diffraction. The coordination geometry is pseudo square pyramidal, with the nitrogen and sulfur donor atoms forming a square base and the oxo ligand at the apex. The deprotonated sulphonate group has a syn conformation with respect to the oxo ligand. The renal clearances of $[^{99m}TcO(MAG_2\text{-}AMS)]^{2-}$ and $[^{99m}TcO(MAG_2\text{-}AMP)]^{3-}$ were similar in rats and suggest that the difference in total charge between the $SO_3^-$ and $PO_3^{2-}$ groups is not important to renal clearance. However, their renal clearances were 40-50% less than that of $[^{99m}TcO(MAG_3)]^{2-}$ suggesting that the size and shape of the large tetrahedral $SO_3^-$ and $PO_3^{2-}$ groups of $[^{99m}TcO(MAG_2\text{-}AMS)]^{2-}$ and $[^{99m}TcO(MAG_2\text{-}AMP)]^{3-}$ inhibit recognition by the renal transport system compared to the small planar $CO_2^-$ group of $[^{99m}TcO(MAG_3)]^{2-}$.

**Introduction**

Mercaptoacetyltriglycine ($MAG_3H_5$) forms complexes with Re and Tc that are substrates of the renal transport system for organic anions. In fact, derivatives of $[^{99m}TcO(MAG_3)]^{2-}$ and $[^{186}ReO(MAG_3)]^{2-}$ ($MAG_3$ = penta-anionic form of $MAG_3H_5$) are being used to radiolabel antibodies in investigational cancer diagnosis and therapy, in part because the radiolabeled catabolites and metabolites are rapidly excreted by the kidneys.[1,2] $[^{99m}TcO(MAG_3)]^{2-}$ is also used in nuclear medicine to evaluate renal tubular function. However, the renal clearance of $[^{99m}TcO(MAG_3)]^{2-}$ is only 50-60% that of radioiodinated $[^{131}I]$-o-iodohippurate (OIH)[3] which is also used clinically to evaluate renal function. Since therapeutic applications for $^{186}Re$ and $^{188}Re$ radiolabeled antibodies are under investigation and $^{99m}Tc$ is the preferred diagnostic radionuclide in nuclear medicine procedures, it is important to develop new ligands that not only form Re and Tc chelates but are also superior substrates for the renal anion transport system.

Organic sulphonates are also substrates for the renal anion transport system. A sulphonate group is similar to a carboxylate group in that both are mono-anionic oxo acids at physiological pH and both serve as the primary recognition site for renal tubular transport. However, a sulphonate is a large tetrahedral group while a carboxylate is a smaller planar group. The charge on a sulphonate group is also more diffuse than that of a carboxylate group because the sulphonate has an additional oxo group participating in electron delocalization.

Previously, Nosco and coworkers[4] reported that the total clearance of $[^{99m}TcO(MAG_2\text{-}taurine)]^{2-}$ ($MAG_2$ = mercaptoacetylglycylglycyl) was slower than that of $[^{99m}TcO(MAG_3)]^{2-}$ in the rat. (Renal clearance was not reported.) Hepatobiliary excretion was evidently significant with the taurine analogue. However, the reasons for the differences in the biological properties of $[^{99m}TcO(MAG_3)]^{2-}$ and $[^{99m}TcO(MAG_2\text{-}taurine)]^{2-}$ were unclear because the tauryl residue in $[^{99m}TcO(MAG_2\text{-}taurine)]^{2-}$ is a β-amino sulphonic acid. The renal clearance of $[^{99m}TcO(MAG_2\text{-}\beta\text{-}alanine)]$ is also inferior to that of $[^{99m}TcO(MAG_3)]^{2-}$.[5]

In an effort to determine the properties that best facilitate rapid renal transport, two derivatives of $MAG_3H_5$ were synthesized. Mercaptoacetylglycylglycylaminomethanesulphonic acid ($MAG_2$-$AMSH_5$) was designed to determine the effect of the differences between a carboxylate and sulphonate group (size, shape and charge distribution) on renal clearance. However, the presence of amine and sulphonic acid groups on the same carbon presented synthetic problems which required a new synthetic approach. Mercaptoacetylglycylglycylaminomethylphosphonic acid ($MAG_2$-$AMPH_6$) was also synthesized to determine if a difference in the formal charge of the anionic group had an effect on renal clearance. The phosphonate group is similar to a sulphonate group in size and shape but it is dianionic at physiological pH. Like $MAG_3H_5$, these ligands form $[Tc(V)O]^{3+}$ and $[Re(V)O]^{3+}$ complexes in which the mercapto and amide donor groups are deprotonated; the terminal anionic groups are also fully deprotonated at physiological pH. Comparisons between $[^{99m}TcO(MAG_2\text{-}AMS)]^{2-}$ ($MAG_2$-AMS = penta-anionic form of $MAG_2$-$AMSH_5$), $[^{99m}TcO(MAG_2\text{-}AMP)]^{3-}$ ($MAG_2$-AMP = hexa-anionic form of $MAG_2$-$AMPH_6$) and $[^{99m}TcO(MAG_3)]^{2-}$ are straightforward because in all three complexes the terminal residues are α-amino acids.

The renal clearances of the $^{99m}Tc$ complexes with the new ligands have been measured in rats.[6] The renal clearances of the sulphonate, $[^{99m}TcO(MAG_2\text{-}AMS)]^{2-}$, and phosphonate, $[^{99m}TcO(MAG_2\text{-}AMP)]^{3-}$, complexes are comparable suggesting that for both complexes the stereoelectronic interaction with the renal anion transport receptor is similar. A representative Re(V) oxo complex was prepared with the sulphonate ligand for characterization and the solid-state structure of $Na_2[ReO(MAG_2\text{-}AMS)]\cdot 3H_2O$ was determined by X-ray diffraction methods. The structure of $[ReO(MAG_2\text{-}AMS)]^{2-}$ is important because the terminal anionic group ($SO_3^-$) (which is expected to be conformationally flexible in solution) is syn to the oxo ligand. A syn conformation is generally believed to be important for efficient renal clearance since *syn*-$[^{99m}TcO(map)]^{2-}$ (map = penta-anionic form of 2,3-bis(mercaptoacetamido)propanoate) is excreted at twice the rate of *anti*-$[^{99m}TcO(map)]^{2-}$ [7] (The carboxylate groups of the $[^{99m}TcO(map)]^{2-}$ complexes are bound to chelate ring carbons and do not show conformational flexibility.) Although $[Ph_4As][^{99}TcO(MAG_3H)]$ ($MAG_3H$ = tetra-anionic form of $MAG_3H_5$ in which the carboxyl group is protonated) has a syn conformation in the solid-state the structural details have not been reported.[8] Others, $[Bu_4N][ReO(MAG_3H)]$[9] and $[Ph_4P][ReO(MAG_2\text{-}oABAH)]$[10] ($MAG_2$-ABAH = tetra-anionic form of mercaptoacetylglycylglycylaminobenzoic acid), adopt anti conformations in the solid-state. Furthermore, the structure of $[ReO(MAG_2\text{-}AMS)]^{2-}$ is unique in that the terminal anionic group ($SO_3^-$) is in its deprotonated, physiologically relevant from. All other $MO(N_3S)$ and $MO(N_2S_2)$ (M = Re, $^{99}Tc$) complexes for which crystal data are available have protonated carboxyl groups.[8-11]

### Materials and Methods

NMR spectra were obtained at 300 MHz for $^1H$ and 75 MHz for $^{13}C$ with a General Electric QE-300 spectrometer. The spectra were recorded in $Me_2SO\text{-}d_6$ or $D_2O$. Chemical shifts (ppm) in $Me_2SO\text{-}d_6$ were referenced to the solvent peak 2.49 ppm ($^1H$) and 39.9 ppm ($^{13}C$) vs. TMS (tetramethylsilane). Chemical shifts in $D_2O$ were referenced to dioxane 3.53 ppm ($^1H$) and 66.5 ppm ($^{13}C$) vs TMS. Elemental analyses were performed by Atlantic Microlabs Inc., Atlanta, GA. FTIR spectra were recorded with a Bruker IFS 66 instrument. Yields are based on purified, air-dried material.

### Ligand Synthesis.

Phthaloylglycyl chloride[12] (PGC) mp 83-85 °C (lit. 83-85 °C), succinimydyl-N-(S-

benzoylthioacetate)[13] (SBzMA-OSucc) $^1$H NMR ($Me_2SO$-$d_6$): 7.93 (d, 2H, SBzH), 7.69 (t, 1H, SBzH,), 7.56 (t, 2H, SBzH), 3.89 (s, 2H, $SCH_2$), and succinimidyl-N-(S-benzoylthioacetyl)glycinate[8] (SBzMAG-OSucc) $^1$H NMR ($Me_2SO$-$d_6$): 8.87 (t, 1H, CONH), 7.93 (d, 2H, SBzH), 7.71 (t, 1H, SBzH), 7.57 (t, 2H, SBzH), 4.30 (d, 2H, $NCH_2$), 3.89 (s, 2H, $SCH_2$), 2.80 (s, 4H, $COCH_2CH_2CO$) were prepared by literature methods.

**Glycylaminomethanesulphonic acid (1).** Aminomethanesulphonic acid (15.3 g, 0.14 mol) and MgO (2.8 g, 0.07 mol) were suspended in $H_2O$ (300 ml) and cooled to 5 °C. PGC (15.7 g, 0.07 mol) in dioxane (75 ml) was added dropwise to the aqueous suspension over 45 min. After the reaction mixture was stirred at room temperature for 1 h, it was brought to pH 1 with concentrated HCl. The solvent was evaporated and the residue was crystallized from EtOH to give phthaloylglycyl-aminomethanesulphonic acid (13.0 g, 0.04 mol) which was deprotected by heating at reflux in alcoholic hydrazine (0.2 M, 300 ml) for 4 h. After cooling to room temperature, the solid material was collected and dissolved in $H_2O$. The solution was filtered to remove any remaining solid. Addition of acetone and allowing the solution to stand at 5 °C overnight afforded crystals. Yield: 4.4 g (38%). Anal. Cacld for $C_3H_8N_2O_4S$: C, 21.43; H, 4.80; N. 16.66. Found: C, 21.48; H, 4.79; N, 16.56. $^1$H NMR ($Me_2SO$-$d_6$): 8.71 (t, 1H CONH), 7.94 (br, 3H, NH), 3.93 (d, 2H, $NCH_2$), 3.54 (s, 2H, $SCH_2$).

**Na[N-(S-Benzoylthioacetyl)glycylglycylaminomethanesulphonate] ·1/2$H_2O$ (2).** 1 (2.0 g, 0.01 mol) was dissolved in 80% $MeOH/H_2O$ (100 ml) by warming and adjusting the pH to 7 with 1 N NaOH. SBzMAG-OSucc (4.0 g, 0.01 mol) was added to the solution and the solution was heated at reflux for 2.5 h. The reaction mixture was left at room temperature overnight. A white solid was collected and washed with hot $CH_3CN$. Yield 2.5 g (50%). Anal. Cacld for $C_{14}H_{17}N_3NaO_{7.5}S$: C, 38.71; H, 3.95; N, 9.67. Found: C, 38.70; H, 4.79; N, 9.69. $^1$H NMR ($Me_2SO$-$d_6$): ppm 8.49 (t, 1H CONH), 8.22 (t, 1H CONH), 8.11 (t, 1H CONH), 7.93 (d, 2H, SBzH), 7.70 t, 1H, SBzH), 7.56 (t, 2H, SBzH), 3.92 (d, 2H, $NCH_2$), 3.87 (s, 2H, $SCH_2$), 3.76 (overlapping d, 4H, $NCH_2$). $^{13}$C NMR ($Me_2SO$-$d_6$): 190.57 (CO), 169.04 (CO), 168.49 (CO), 167.41 (CO), 136.41 (SBzC), 134.30 (SBzC), 129.38 (2C, SBzC), 127.12 (2C, SBzC), 55.50 ($NCH_2$), 42.65 ($NCH_2$), 42.08 ($NCH_2$), 32.68 ($SCH_2$).

**Succinimidyl-N-(S-benzoylthioacetyl)glycylglycinate (3).** SBzMA-OSucc (7.3 g, 25 mmol) was dissolved in warm EtOH (100 ml). Glycylglycine (3.3 g, 25 mmol) was dissolved in $H_2O$ (30 ml) and the pH of the solution was brought to 7 with 1 N NaOH. The aqueous solution was added to the alcoholic solution and heated at reflux for 2 h. The solution was left at room temperature overnight. A white solid was collected and washed with MeOH. Yield 5.1 g (66%) for N-(S-benzoylthioacetyl)glycylclycine ($SBzMAG_2$). $SBzMAG_2$ (5.0 g, 16 mmol) was dissolved in THF (200 ml) along with N-hydroxysuccinimide (1.9 g, 16 mmol). The solution was cooled to 0 °C. Dicyclohexylcarbodiimide (4.1 g, 20 mmol) in THF (80 ml) and added dropwise over 30 min. The reaction mixture was stirred for an additional 2 h at 0 °C and then left at room temperature overnight. Dicyclohexylurea was removed by filtration and the filtrate evaporated to dryness. The residue was crystallized from ethyl acetate and iPrOH. Yield: 5.3 g (81%; 53% overall). Anal. Cacld for $C_{17}H_{17}N_3O_7S$: C, 50.12; H, 4.21; N, 10.31. Found: C, 50.14; H, 4.25; N, 10.23. $^1$H NMR ($Me_2SO$-$d_6$): 8.57 (t, 2H, CONH), 7.93 (d, 2H, SBzH), 7.70 (t, 1H, SBzH), 7.56 (t, 2H, SBzH), 4.28 (d, 2H, $NCH_2$), 3.88 (s, 2H, $SCH_2$), 3.89 (d, 2H, $NCH_2$), 2.80 (s, 4H, $C(O)CH_2CH_2CO$).

**Na[N-(S-Benzoylthioacetyl)glycylglycylaminomethylphosphonate]·2$H_2O$ (4).** Aminomethylphosphonic acid (0.5 g, 4.5 mmol) was dissolved in $H_2O$ (10 ml) and the pH of the solution was brought to 7 with 1 N NaOH. $SBzMAG_2$-OSucc (1.3 g, 3.2 mmol) in MeOH (20 ml) was added and the reaction mixture was stirred at room temperature for 5 h. The solution was filtered and acetone (100 ml) was added to the filtrate. The precipitate was collected and dissolved in 67% $MeOH/H_2O$ (30 ml) and re-precipitated with acetone (100 ml). Yield 0.5 g (35%). Anal. Cacld for $C_{14}H_{21}NaN_3O_9PS$: C, 36.45; H, 4.59; N, 9.11. Found: C, 36.41; H, 4.45; N, 9.47. $^1$H NMR ($D_2O$): 7.76 (d, 2H, SBzH), 7.50 (t, 1H, SBzH), 7.38 (t, 2H, SBzH), 3.81 (s, 2H, $CH_2$), 3.76 (s, 2H, $CH_2$), 3.72 (s, 2H, $CH_2$),3.19 (d, 2H, $CH_2P$). $^{13}$C NMR ($D_2O$): 194.27 (CO), 171.82 (CO), 171.72 (CO), 170.61 (CO), 135.44 (SBzC), 134.62 (SBzC), 128.99 (2C, SBzC), 127.20 (2C, SBzC), 42.88 ($NCH_2$), 42.36 ($NCH_2$), 37.32 (d, J = 146 Hz, $CH_2P$), 32.54 ($SCH_2$).

**Synthesis of Rhenium(V) Complexes.**

**$ReOCl_3(Me_2S)(OPPh_3)$ (5):** This complex was prepared by the method of Grove and Wilkinson.[14a] FTIR in KBr: 992 $cm^{-1}$ [Re=O], 1139 $cm^{-1}$ [P-O] (lit. 992 $cm^{-1}$ [Re=O], 1140 $cm^{-1}$ [P-O]).[14b]

**$Na_2$[ReO(mercaptoacetylglycylglycylaminomethanesulphonate)]·$3H_2O$ (6).** **2** (0.37 g, 0.85 mmol) was dissolved in 63% MeOH/$H_2O$ (16 ml) and the pH of the solution was brought to 8 with 1 N NaOH. **5** (0.55 g, 0.85 mmol) was added to the solution to give a green suspension which was heated to 65-70 °C. As the reaction proceeded, a pH of 8 was maintained by dropwise addition of 1 N NaOH. The green suspension gradually cleared to give an orange solution after 1 h. The solution was cooled to room temperature, filtered and extracted twice with $CHCl_3$. Ammonium hydroxide (5 ml) was added to the solution along with acetone (100 ml) and the resulting solution left to stand at 5 °C overnight. Orange microcrystals were collected and washed with acetone. Yield 0.31 g (61%). X-ray quality crystals were obtained by slow diffusion of EtOH into a saturated solution of the complex in $H_2O$. Anal. Cacld for $C_7H_{14}Na_2N_3O_{10}ReS_2$: C, 14.10 H, 2.37; N, 7.04. Found: C, 14.23 H, 2.40; N, 7.03. $^1H$ NMR ($Me_2SO$-$d_6$): 5.48, 4.39 (dd, 2H, J = 12 Hz, $NCH_2$), 4.53 4.08 (dd, 2H, J = 18 Hz, $NCH_2$), 4.13, 4.06 (dd, 2H, J = 18 Hz, $NCH_2$), 3.75, 3.63 (dd, 2H, J = 17 Hz, $SCH_2$). $^{13}C$ NMR: 192.43 (CO), 191.38 (CO), 188.58 (CO), 69.14 ($NCH_2$), 56.18 ($NCH_2$), 53.19 ($NCH_2$), 38.76 ($SCH_2$). FTIR in KBr: 972 $cm^{-1}$ [Re=O].

**X-ray Crystallography.**

An orange prism of **6** having approximate dimensions of 0.26 x 0.42 x 0.58 mm was used for data collection on a Siemens P4 diffractometer. The crystal system and cell dimensions were determined by least-squares refinement of 30 centered reflections, 10 of which were > 24° in 2θ. Two check refections were measured every 48 reflections and there was no significant deviation in intensities. Intensities were corrected for Lorentz and monochromator polarization effects and a semi-empirical absorption correction was applied based on azimuthal scans of 8 reflections. Systematic absences were consistent with the unique space group Pbca. The structure was solved by Patterson methods and all non-hydrogen atoms were refined anisotropically by full-matrix least-squares procedures using SHELXTL PLUS (VMS). The water hydrogen atoms were located from difference maps and the methylene hydrogen atoms were generated at calculated ($d$(C-H) = 0.96 Å) positions. The hydrogen atoms were constrained using a riding model with isotropic thermal parameters fixed at 0.08. Crystallographic data are summarized in Table I.

### Results

The synthesis of S-benzoyl (SBz) protected [SBzMAG$_2$-AMPH$_4$]$^-$ (**2**) using a standard activated ester coupling procedure[10] was moderately successful (33% yield) at room

**Table I.** Crystallographic Data for $Na_2$[ReO(MAG$_2$-AMS)]·$3H_2O$ (**6**).

| | |
|---|---|
| chemical formula | $C_7H_{14}Na_2N_3O_{10}ReS_2$ |
| fw | 596.5 |
| space group | Pbca |
| Z | 8 |
| a (Å) | 18.039 (3) |
| b (Å) | 9.774 (2) |
| c (Å) | 18.474 (3) |
| volume (Å$^3$) | 3257.2 (10) |
| $d_{calcd}$ (g/cm$^3$) | 2.43 |
| μ (mm$^{-1}$) | 7.91 |
| λ | 0.71073 Å (Mo Kα) |
| T (K) | 298 |
| min./max. transmission | 0.0005 /0.0066 |
| R (%) | 5.97 |
| $R_w$ (%) | 6.96 |
| goodness-of-fit | 1.86 |

temperature. Heating the reaction gave product that contained an impurity (by NMR) that was difficult to remove. The synthesis of [SBzMAG$_2$-AMSH$_3$]$^-$ (**4**) was completely unsuccessful using the activated ester coupling procedure. α-Amino sulphonic acids decompose to aldehyde, sulfur dioxide and ammonia under acidic and alkaline conditions apparently because the free electron pair on the nitrogen participates in the formation of an intermediate imine followed by loss of a proton and hydrogen sulfite.[15]

$$NH_2\text{-}CH(R)\text{-}SO_3H \rightarrow NH{=}CH(R) + SO_3H^- + H^+ \rightarrow R\text{-}CH{=}O + SO_2 + NH_3$$

Undoubtedly, the tendency of α-amino sulphonic acids to undergo such an electronic shift reduces their nucleophilicity and the lack of any reactiviy between aminomethanesulphonic acid (AMS) and the relatively stable succinimidyl ester (SBzMAG$_2$-OSucc) was unanticipated but not surprising.

N-(carbobenzoxy)glycine and AMS were coupled using isobutylchloroformate by Frankel and Moses[16] in the late 1950's (43% yield). However, removal of the protecting group by treating with HBr to give glycylaminomethanesulphonic acid (**1**) resulted in an overall yield of 8%. In the present study, phthaloylglycyl chloride (PGC) and AMS were coupled based on the method of Sheehan and Frank.[12] The phthaloyl protecting group was readily removed with hydrazine giving **1** in an overall yield of 38%. **1** was then coupled with SBzMAG-OSucc by the usual activated ester procedure to give [SBzMAG$_2$-AMSH$_3$]$^-$. Both [SBzMAG$_2$-AMSH$_3$]$^-$ and [SBzMAG$_2$-AMPH$_4$]$^-$ were isolated as mono-sodium salts.

[SBzMAG$_2$-AMSH$_3$]$^-$ (**2**) reacted smoothly with ReOCl$_3$(Me$_2$S)(OPPh$_3$) in aqueous methanol resulting in a clear orange solution from which Na$_2$[ReO(MAG$_2$-AMS)] (**6**) was isolated. The presence of a Re=O group was confirmed by a strong absorption in the FTIR spectrum at 972 cm$^{-1}$.[9-11] The coupling between geminal protons in the $^1$H NMR spectrum is consistent with a square-pyramidal coordination environment.[10] The $^{13}$C NMR signals are shifted significantly downfield from the corresponding signals in the free ligand. Similar downfield shifts were observed for the $^{13}$C NMR signals of the ortho, meta and para isomers of [ReO(MAG$_2$-ABAH)]$^-$ (MAG$_2$-ABAH = tetra-anionic form of mercaptoacetylglycylglycylaminobenzoic acid). [10]

[SBzMAG$_2$-AMPH$_4$]$^-$ (**4**) also reacted smoothly with ReOCl$_3$(Me$_2$S)(OPPh$_3$) in aqueous methanol resulting in a clear orange solution. However, the product could only be obtained in poor yield as a crude oil. The FTIR spectrum in KBr of the crude product showed a strong band at 975 cm$^{-1}$ characteristic of a Re=O stretch.[9-11] The $^1$H NMR spectrum included signals similar to those observed in the spectrum of Na$_2$[ReO(MAG$_2$-AMS)]·3H$_2$O (**6**) except that two of the signals attributable to the geminal NC$\underline{H_2}$P protons were split into triplets due to coupling with each other and phosphorus.

The structure of Na$_2$[ReO(MAG$_2$-AMS)]·3H$_2$O (**6**) was determined by X-ray diffraction. A perspective drawing of the dianion of **6** is presented in Figure 1. Final atomic coordinates are listed in Table II and selected bond distances and angles are given in Table III. The coordination geometry is square pyramidal with Re displaced 0.75 Å out of the ligand coordination plane (S(1), N(1), N(2), N(3), mean deviation = 0.02 Å) toward the apical oxo ligand. The Re bond distances and angles are similar to those in the structures of other Re(V)O(N$_3$S) species: [Bu$_4$N][ReO(MAG$_3$H)],[9] [Ph$_4$P][ReO(MAG$_2$-pABAH)]·H$_2$O,[10] and [Ph$_4$P][ReO(MAG$_2$-oABAH)].[10]

The sulphonate group in **6** is is deprotonated and adopts a syn conformation with respect to the oxo ligand. Both the protonation state and the conformation of the terminal group are important for the renal anion transport system because (1) the terminal group, in its anionic form, serves as the primary recognition site for renal tubular transport and (2) the oxo ligand is considered to be a secondary recognition site (at least in the related carboxylate species).[11,17] Since *syn*-[$^{99m}$TcO(map)]$^{2-}$ is excreted at twice the rate of *anti*-[$^{99m}$TcO(map)]$^{2-}$ [7] simultaneous recognition of the primary anionic group and the oxo ligand may be most effective when the primary anionic and secondary (oxo) recognition sites are on the same side of the metal coordination plane. [11] The structure of **6** is the first example of a Re(V)ON$_3$S complex that satisfies the charge and conformational criteria for renal tubular transport in the solid-state. In the reported crystal structures of other renal agents {[Bu$_4$N][ReO(MAG$_3$H)],[9] [Ph$_4$P][ReO(MAG$_2$-pABAH)]·H$_2$O,[10] [PPh$_4$][ReO(MAG$_2$-oABAH)],[10] [Ph$_4$As][$^{99}$TcO(mapH)],[11]

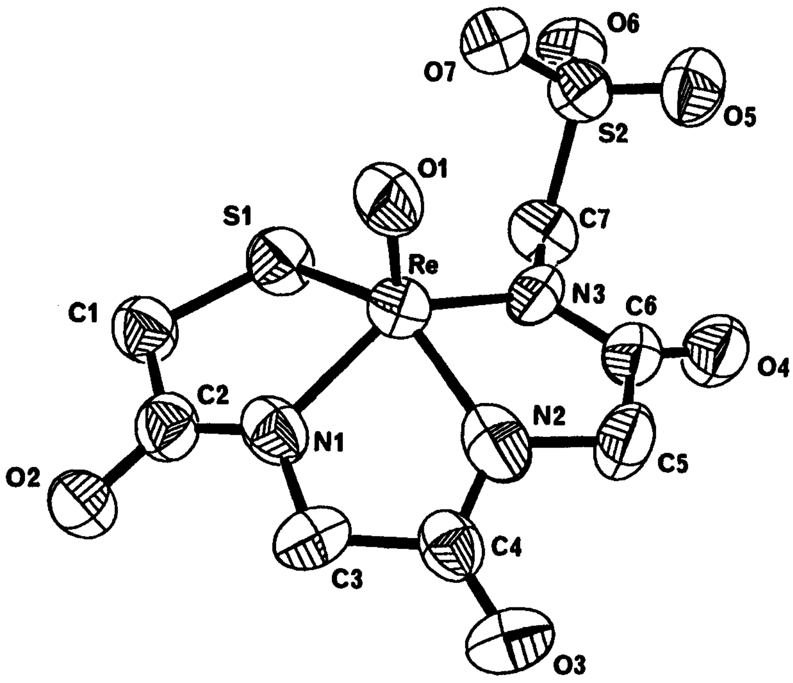

**Figure 1.** Perspective drawing of $Na_2[ReO(MAG_2\text{-}AMS)]\cdot 3H_2O$ (dianion of **6**) with 50%. probability for the thermal ellipsoids.

**Table II.** Atomic Coordinates (x$10^4$) and Equivalent Isotropic Displacement Coefficients (Å$^2$ x $10^3$) for $Na_2[ReO(MAG_2\text{-}AMS)]\cdot 3H_2O$ (**6**).

| atom | x | y | z | U(eq) |
|---|---|---|---|---|
| Re | 8547(1) | 1935(1) | 4256(1) | 52(1) |
| S(1) | 7540(2) | 710(3) | 4629(2) | 62(1) |
| S(2) | 8499(2) | -1090(3) | 2779(2) | 57(1) |
| N(1) | 8400(6) | 2876(10) | 5212(6) | 54(3) |
| N(2) | 9595(6) | 2322(12) | 4567(6) | 61(4) |
| N(3) | 9087(5) | 195(11) | 3938(6) | 56(3) |
| O(1) | 8282(5) | 2941(9) | 3566(5) | 67(3) |
| O(2) | 7683(5) | 3543(10) | 6142(5) | 63(3) |
| O(3) | 10346(5) | 3529(10) | 5303(5) | 67(3) |
| O(4) | 10242(5) | -717(10) | 3719(6) | 76(4) |
| O(5) | 8154(5) | -2419(10) | 2674(5) | 64(3) |
| O(6) | 9160(5) | -888(11) | 2351(5) | 73(3) |
| O(7) | 7972(5) | 18(8) | 2700(5) | 60(3) |
| C(1) | 7194(7) | 1835(13) | 5329(8) | 61(4) |
| C(2) | 7772(8) | 2845(14) | 5588(7) | 60(5) |
| C(3) | 9026(7) | 3689(16) | 5458(7) | 62(5) |
| C(4) | 9733(8) | 3185(13) | 5106(7) | 57(5) |
| C(5) | 10176(8) | 1506(17) | 4228(8) | 66(5) |
| C(6) | 9851(7) | 263(16) | 3929(7) | 62(5) |
| C(7) | 8756(7) | -1084(13) | 3716(7) | 58(4) |
| Na(1) | 6714(3) | 307(6) | 3024(3) | 69(2) |
| Na(2) | 8103(3) | 2250(5) | 2272(3) | 64(2) |
| O(8) | 6653(5) | -512(9) | 1804(5) | 67(3) |
| O(9) | 5489(5) | 273(10) | 3356(5) | 74(3) |
| O(10) | 9409(6) | 2239(11) | 2384(6) | 82(4) |

* Equivalent isotropic U defined as one third of the trace of the orthogonalized Uij tensor

**Table III.** Selected Bond Distances (Å) and Angles (deg) for $Na_2[ReO(MAG_2\text{-}AMS)]\cdot 3H_2O$ (6).

| | | | |
|---|---|---|---|
| | Bond distances (Å) | | |
| Re-O(1) | 1.680(9) | Re-N(2) | 2.011(10) |
| Re-S(1) | 2.282(3) | Re-N(3) | 2.046(10) |
| Re-N(1) | 2.009(10) | | |
| | Bond angles (deg) | | |
| S(1)-Re-O(1) | 108.0(3) | N(3)-Re-O(1) | 113.8(4) |
| S(1)-Re-N(1) | 82.5(3) | Re-S(1)-C(1) | 99.8(4) |
| S(1)-Re-N(2) | 139.5(3) | Re-N(1)-C(2) | 124.1(9) |
| S(1)-Re-N(3) | 91.7(3) | Re-N(1)-C(3) | 115.0(8) |
| N(1)-Re-N(2) | 77.7(4) | Re-N(2)-C(4) | 120.7(9) |
| N(1)-Re-N(3) | 134.1(4) | Re-N(2)-C(5) | 116.7(9) |
| N(2)-Re-N(3) | 78.0(4) | Re-N(3)-C(6) | 116.0(9) |
| N(1)-Re-O(1) | 111.2(4) | Re-N(3)-C(7) | 127.2(8) |
| N(2)-Re-O(1) | 112.0(5) | | |

$[Ph_4P][^{99}TcO(mapH)]$[11] and $[Ph_4As][ReO(mapH)]$]}[11] the terminal carboxyl groups are protonated and the orientation of the carboxyl group is anti to the oxo ligand in $[Bu_4N][ReO(MAG_3H)]$[9] and $[PPh_4][ReO(MAG_2\text{-}oABAH)]$]}.[10] However, although the solid-state structure of **6** is in its deprotonated, physiologically relevant form, the observed syn conformation could be due to lattice interactions. Therefore, we examined possible interactions in the solid.

Within the crystal lattice there is an O(1), O(5), O(7), O(8) bridging network (Figure 2) that creates four $Na^+$ channels with interatomic Na-Na distances of 3.3-3.4 Å running parallel to the b axis. Na(1) is surrounded in a pseudo-octahedral manner by S(1), O(7), two water oxygen atoms (O(8) and O(9)), and two oxygen atoms from symmetry related dianions (O(1a) symmetry position (1.5-x, -0.5+y, z) and O(5a) symmetry position (1.5-x, 0.5+y, z)). Na(2) is also surrounded in a pseudo-octahedral manner by O(1), O(7), two water oxygen atoms (O(10) and O(8a) symmetry

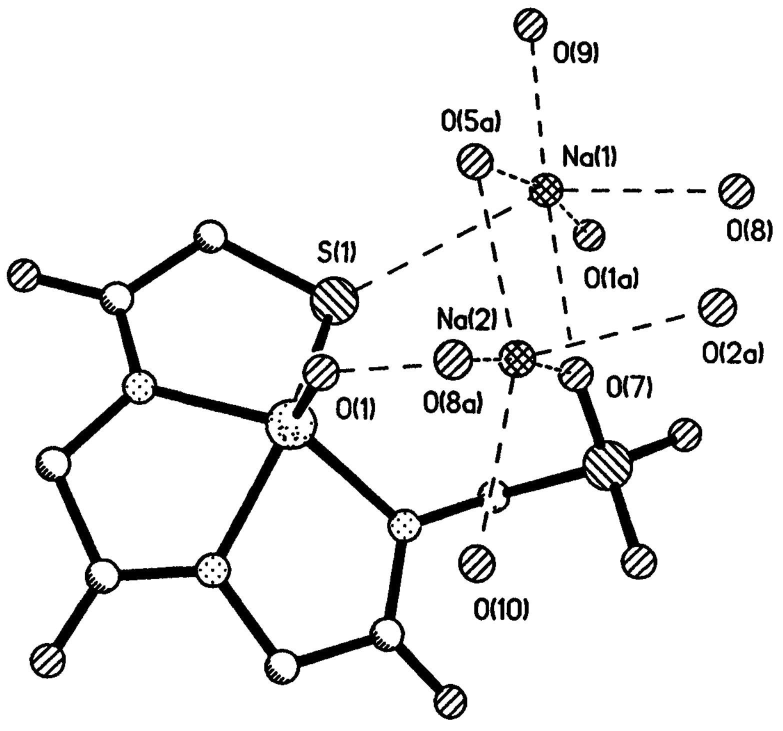

**Figure 2.** $Na^+$ coordination in the crystal lattice of $Na_2[ReO(MAG_2\text{-}AMS)]\cdot 3H_2O$ (6).

position (1.5-x, 0.5+y, z)), and two oxygen atoms from symmetry related dianions (O(2a) symmetry position (x, 0.5-y, -0.5+z) and O(5a) symmetry position (1.5-x, 0.5+y, z)). Na(1) and Na(2) are bridged by O(7) and O(5a) with a Na-Na distance of 3.4 Å. Na(1) is bridged to Na(2a) symmetry position (1.5-x, -0.5+y, z) by O(1) and Na(2) is bridged to Na(1a) symmetry position (1.5-x, 0.5+y, z) by O(8) with Na-Na distances of 3.3 Å. The syn conformation of the dianion is evidently important in the solid state since Na(2) is coordinated by both O(1) and O(7) and three out of the four bridging oxygen atoms are sulphonate and oxo species.

### Discussion

The predominant difference between $[MO(MAG_2\text{-}AMS)]^{2-}$ and $[MO(MAG_3)]^{2-}$ (M = Tc. Re) appears to be the size and shape of the terminal anionic group ($SO_3^-$ vs $CO_2^-$) based on solid-state models. The bond distances and angles associated with the metal coordination spheres of $Na_2[ReO(MAG_2\text{-}AMS)]\cdot 3H_2O$ and $[Bu_4N][ReO(MAG_3H)]$[9] are not significantly different and those of $[Ph_4As][^{99}TcO(MAG_3H)]$[8] are comparable. Although there is a difference in the conformation of the terminal anionic group of $Na_2[ReO(MAG_2\text{-}AMS)]\cdot 3H_2O$ (and $[Ph_4As][^{99}TcO(MAG_3H)]$) and $[Bu_4N][ReO(MAG_3H)$, there is evidence that suggests the conformation is influenced by lattice interactions involving coordination of both the oxo ligand (O(1)) and the sulphonate oxygen atoms (O(5) and O(7)) to the $Na^+$ ions in an extended bridging network. Furthermore, these complexes should be conformationally flexible in solution. $[^{99m}TcO(MAG_2\text{-}AMS)]^{2-}$ and $[^{99m}TcO(MAG_2\text{-}AMP)]^{3-}$ are also expected to be structurally similar since a phosphonate group is similar in size and shape to a sulphonate group,.

The renal clearances (in rats) of $[^{99m}TcO(MAG_2\text{-}AMS)]^{2-}$ and $[^{99m}TcO(MAG_2\text{-}AMP)]^{3-}$ were approximately equal[6] but showed a 40-50% decrease in renal clearance compared to $[^{99m}TcO(MAG_3)]^{2-}$. These results suggest that the difference in total charge between the sulphonate group and phosphonate group is not important for renal clearance. If the total charge had been an important parameter, a significant difference in the clearance rates for $[^{99m}TcO(MAG_2\text{-}AMS)]^{2-}$ and $[^{99m}TcO(MAG_2\text{-}AMP)]^{3-}$ would have been observed. However, the similar decrease in the renal clearance of $[^{99m}TcO(MAG_2\text{-}AMS)]^{2-}$ and $[^{99m}TcO(MAG_2\text{-}AMP)]^{3-}$ compared to $[^{99m}TcO(MAG_3)]^{2-}$ suggest that the size and shape of a small planar $CO_2^-$ group is more easily recognized by the renal anion transport system than the larger tetrahedral $SO_3^-$ and $PO_3^{2-}$ groups.

**Acknowledgement.** We gratefully acknowledge financial support from the National Institutes of Health (Grant No. DK38842-04).

### References

1. Axworthy, D. B.; Kasina, S.; Rao, T. N.; Srinivasin, A.; Fritzberg, A. R. *J. Nucl. Med.* 1991, **5**, 915 (abstract).
2. Axworthy, D. B.; Fu-Min, S.; Vanderheyden, J-L.; Srinivasin, A.; Fitzner, J.; Glaster, J.; Beaumier, P.; Fritzberg, A. R. *J. Nucl. Med.* 1991, **5**, 915 (abstract).
3. (a) Taylor, A.; Ziffer, J. A.; Steves, A.; Eshima, D.; Delaney, V. B.; Welchel, J. D. *Radiology* 1989, **170**, 721. (b) Jafri, R. A.; Britton, K. E.; Nimmon, C.C. et al. *J. Nucl. Med.* 1988, **29**, 147. (c) Russel, C. D.; Thorstad, B.; Yester, M. V. et al. *J. Nucl. Med.* 1988, **29**, 1189.
4. Nosco, D.L; Coveney, J. R.; Helling, D. E.; MacDonald, J. R.; Rajagopalan, R.; Wallach, S. M. *J. Nucl. Med.* 1989, **30**, 937 (abstract).
5. Dennis Eshima, Ph.D., Emory University, Atlanta, GA. Unpublished results.
6. Hansen, L.; Marzilli, L. G.; Eshima, D.; Malveaux, G.; Folks, R.; Taylor, A., Jr. *J. Nucl. Med.* submitted.
7. Klingensmith, W. C., III; Fritzberg, A. R.; Spitzer, A. M. *J. Nucl. Med.* 1984, **25**, 42.
8. (a) Nosco, D. L.; Manning, R. G. *J. Nucl. Med.* 1986, **27**, 939 (abstract)
   (b) Dennis Nosco, Ph. D., Mallinkrodt, Inc., unpublished data (cited with permission).
9. Rao, T. N.; Adhikesavalu, D.; Camerman, A.; Fritzberg, A. R. *Inorg. Chim. Acta.* 1991, **180**, 63.

10. Hansen, L.; Cini, R.; Taylor, A., Jr.; Marzilli, L. G. *Inorg. Chem.* 1992, **31**, 2801.
11. Rao, T. N.; Adhikesavalu, D.; Camerman, A.; Fritzberg, A. R. *J. Am. Chem. Soc.* 1990, **112**, 5798.
12. Sheehan, J. C.; Frank, V. S. *J. Am. Chem. Soc.* 1949, **71**, 1856.
13. Schneider, R. F.; Subramanian, G.; Feld, T. A.; McAfee, J. G.; Zapf-Longo, C.; Palladino, E.; Thomas, F. D. *J. Nucl. Med.* 1984, **25**, 223.
14. (a) Wilkinson, G.; Grove, D. E. *J. Chem. Soc. A.* 1966, 1224. (b) Bryan J. C.; Stenkamp, P. E.; Tulip, H. H.; Mayer, J. M. *Inorg. Chem.* 1987, **26**, 2283.
15. Hinkel, L. E.; Ayling E. E.; Beynon, J. H. *J. Chem. Soc.* 1936, 184.
16. Frankel, M.; Moses, P. *Tetrahedron* 1960, **9**, 289.
17. Despopoulos, A. *J. Theor. Biol.* 1965, **8**, 163.

**Received: June 28, 1993 - Accepted: July 5, 1993 - Received in revised camera-ready form: September 9, 1993**

